# Relationship between *Helicobacter pylori* infection and bone mineral density: a retrospective cross-sectional study

**DOI:** 10.1186/s12876-018-0780-4

**Published:** 2018-04-24

**Authors:** Bo-Lin Pan, Chih-Fang Huang, Seng-Kee Chuah, Jui-Chin Chiang, Song-Seng Loke

**Affiliations:** 1 Department of Family Medicine, Kaohsiung Chang Gung Memorial Hospital and Chang Gung University College of Medicine, 123, Dapi Road, Niaosong District, Kaohsiung, 833 Taiwan; 2 Division of Hepatogastroenterology, Department of Internal Medicine, Kaohsiung Chang Gung Memorial Hospital and Chang Gung University College of Medicine, 123, Dapi Road, Niaosong District, Kaohsiung, 833 Taiwan

**Keywords:** *Helicobacter pylori*, Bone mineral density, body mass index, Dual energy X-ray absorptiometry scan, Osteoporosis

## Abstract

**Background:**

*Helicobacter pylori (H. pylori)* infection can induce individual inflammatory and immune reactions which associated with extra-digestive disorders. Our aim is to investigate the association between *H. pylori* infection and bone mineral density.

**Methods:**

This retrospective cross-sectional study was performed by using the data from the health examination database in a medical center of southern Taiwan in 2013. We investigated the relationship between sex, age, body mass index (BMI), waist circumstance, lipid profile, *H. pylori* infection, the findings of upper gastrointestinal endoscopy and bone mineral density (BMD). Because of nonrandomized assignment and strong confounding effect of age on BMD, the 1:1 propensity score match was applied for age adjustment. The simple and multiple stepwise logistic regression analysis were performed to assess the risk factors of decreased BMD in these well-balanced pairs of participants.

**Results:**

Of the 867 subjects in final analysis with the mean age of 55.9 ± 11.3 years, 381 (43.9%) subjects had *H. pylori* infection, and 556 (64.1%) subjects had decreased BMD. In decreased BMD group, the portion of woman was higher than a normal BMD group (37.2% versus 29.6%, *P* = 0.023), the age was significantly older (59.4 ± 9.8 versus 49.8 ± 11.3, *p* < 0.001) and BMI was significantly lower (24.7 ± 3.5 versus 25.4 ± 3.7, *p* = 0.006) than the normal BMD group. The prevalence of *H. pylori* infection was 39.9% and 46.2% in the normal BMD group and the decreased BMD group respectively (*P* = 0.071). The multivariate analysis which was used for these possible risk factors showed that only advanced age (OR 1.09, 95% CI 1.08–1.11, *P* < 0.001), and low BMI (OR 0.91, 95% CI 0.87–0.95, *P* < 0.001) were independently significantly associated with decreased BMD in this nonrandomized study. In the propensity score-matched participants, the multiple stepwise logistic regression analysis revealed *H. pylori* infection (OR 1.62, 95% CI 1.12–2.35, *P* = 0.011) and low BMI (OR 0.92, 95% CI 0.87–0.97, *P* = 0.001) were independently significantly associated with decreased BMD.

**Conclusions:**

*H. pylori* infection and low BMI were independently significantly associated with decreased BMD in selected propensity score-matched populations after age adjustment.

## Background

Osteoporosis is a silent health problem characterized by decreased bone mineral density (BMD) with a risk of spine and hip fractures. Approximately half of the hip fractures globally result from osteoporosis. According to data from the National Health Insurance Research Database between 1996 and 2010 in Taiwan, the high annual incidence rate of hip fracture was 472.1 per 100,000 patients per year, higher than that in other Asian countries and even in the world. The in-hospital mortality rates were between 0.85 to 2.26% [1]. The spine and hip fractures resulted from osteoporosis could induce patients to become bedridden and needing care. Mortality and disability-associated osteoporosis have significant impact on prognosis and are a burden affecting patients, their families, society, and the health system. Early identification of the risk of decreased BMD and osteoporosis is very important. Previous study has revealed that the risk factors of osteoporosis include age, sex, low body mass index (BMI), steroid use and chronic alcohol consumption [2, 3]. Recently, several gastrointestinal diseases such as inflammatory bowel disease, peptic ulcer disease and atrophic gastritis have been suspected of being risk factors for osteoporosis [4–6].

*Helicobacter pylori* (*H. pylori*) infection is strongly associated with chronic gastritis, peptic ulcers, gastric cancer, and mucosa-associated lymphoid tissue lymphoma [7]. Several studies have reported *H. pylori* also plays a role in extra-digestive disorders including cardiovascular, neurological, skin disease and diabetes mellitus [8].

*H. pylori* infection can induce individual inflammatory and immune reactions, which can regulate bone turnover. Several research studies have reported that *H. pylori* infection is a risk factor of osteoporosis [9, 10], but this finding is controversial in other studies [11, 12]. To our knowledge, only one study has evaluated the relationship between *H. pylori* infection and osteoporosis in Taiwan, but the result was only found in elderly females [10]. Therefore, we aimed to investigate whether *H. pylori* infection was associated with decreased BMD in a general population undergoing routine health examination in Taiwan.

## Methods

### Subjects

This retrospective cross-sectional study was performed by using the data from the health examination database in a medical center of the southern Taiwan. We included subjects who were aged greater than 20 years, had undergone upper gastrointestinal endoscopy with the Campylobacter-like organism test (CLO test) and dual energy X-ray absorptiometry scan (DEXA) from January 2013 to December 2013. Subjects with missing data and gastric cancer were excluded. The study was approved by the Chang Gung Medical Foundation Institutional Review Board (IRB No.: 201701187B0). Since this is a retrospective study, a written consent is waived by an IRB and is deemed unnecessary.

The medical records showed information on sex, age, waist circumstance, lipid profile, *H. pylori* testing, BMD, and the findings of upper gastrointestinal endoscopy. BMI was calculated as the weight in kilograms divided by the height in meters squared. Peptic ulcer or gastro-esophageal reflux disease (GERD) were diagnosed by upper gastrointestinal endoscopy.

### Definition of *H. pylori* infection

The present of *H. pylori* infection was determined by CLO test via upper gastrointestinal endoscopy. CLO test is a rapid diagnostic test and is performed during upper gastrointestinal endoscopy. A biopsy of mucosa from the stomach is placed into the medium consisting of urea and an indicator. If *H. pylori* is present, the urease produced by *H. pylori* converts urea to ammonia, which increases the pH of the medium and then changes its color from yellow to red. In this health examination, all examinations were self-paid, CLO test was performed not only for peptic ulcer, but also performed under subjects request although no peptic ulcer was found by upper gastrointestinal endoscopy.

### Determination of bone mineral density

BMD was measured by DEXA. The *T*-score is the number of standard deviations by which a given measurement differs from the mean for a normal young adult reference population. According to the World Health Organization definition [13], osteoporosis is defined as *T*-score ≤ − 2.5, and the osteopenia is defined as a T-score between − 1 and − 2.5. Decreased BMD in the present study included osteoporosis and osteopenia.

### Statistical analysis

All data are described as the mean ± standard deviation for continuous variables and as numbers and percentages for categorical variables. SPSS software version 19.0 (IBM Corp., Armonk, NY, USA)) was used for the statistical analysis. The characteristics of subjects were compared by *χ*^*2*^-test for the categorical variables and Student’s t-test for the continuous variables. Univariate logistic regression analysis and multivariate logistic regression analysis were conducted to analyze the odds ratio (OR) of significant factors associated with decreased BMD. Besides, to minimize confounding effect of age due to nonrandomized assignment, propensity scores were calculated using a logistic regression model and the covariate, age. A 1:1 matched study group was created by the Greedy method with NCSS software (NCSS 10, NCSS Statistical software, Kaysville, Utah). After adjusting the age, univariate logistic regression analysis and multivariate logistic regression analysis were used to evaluate factors associated with decreased BMD. The strength of association was reported as OR with 95% confidential interval (CI) and *P*-values. All statistical assessments were two-sided and considered significant if *P* < 0.05.

## Results

### Prevalence of *H. pylori* infection and decreased BMD

We enroll 942 subjects who participated in the health examination and underwent biochemistry blood examination, upper gastrointestinal endoscopy with CLO test and the bone mineral density examinations. Seventy-five subjects were excluded due to missing data in some biochemical variables. Of the 867 subjects in final analysis with the mean age of 55.9 ± 11.3 years, 381(43.9%) subjects had *H. pylori* infection, and 556 (64.1%) subjects had decreased BMD. The numbers of female and male subjects were 299 (34.5%) and 568 (65.5%). Table 1 shows the baseline characteristics of all participants.

**Table 1:** Baseline characteristics

	Participants *N* = 867
Sex	
Female n, %	299(34.5%)
Male n, %	568(65.5%)
Age (years old)	55.9 ± 11.3
BMI(Kg/m^2^)	24.9 ± 3.6
Waist circumference(cm)	85.1 ± 10.4
Total cholesterol(mg/dl)	195.7 ± 35.7
HDL cholesterol(mg/dl)	56.5 ± 15.6
Triglyceride(mg/dl)	131.9 ± 84.7
LDL cholesterol(mg/dl)	113.4 ± 32.4
Decreased BMD, n %	556(64.1%)
Peptic ulcer n, %	351(40.5%)
GERD n, %	187(21.6%)
*H. pylori* infection n, %	381(43.9%)

*BMI* body mass index, *LDL* low-density lipoprotein, *HDL* high-density lipoprotein, *BMD* bone mineral density, *GERD* gastro-esophageal reflux disease, H. pylori *Helicobacter pylori*

### Differences between the subjects with normal and decreased BMD

Table 2 compares the characteristics between the normal BMD group and the decreased BMD group. In decreased BMD group, the portion of woman was higher than a normal BMD group (37.2% versus 29.6%, *P* = 0.023), the age was significantly older (59.4 ± 9.8 versus 49.8 ± 11.3, *p* < 0.001) and BMI was significantly lower (24.7 ± 3.5 versus 25.4 ± 3.7, *p* = 0.006) than the normal BMD group. Besides, lipid profiles except for triglyceride were significantly higher than in the normal BMD group. Comparing the gastrointestinal disorder between these two groups, the prevalence of peptic ulcer was significantly higher in the decreased BMD group (*p* = 0.032), but the GERD was lower in the decreased BMD group (*p* = 0.741). The prevalence of *H. pylori* infection was 39.9% and 46.2% in the normal BMD group and decreased BMD group respectively (*P* = 0.071).

**Table 2:** Differences between normal BMD group and decreased BMD group

	Normal BMD *n* = 311	Decreased BMD *n* = 556	*p* value
Sex			0.023*
Male n, %	219(70.4%)	349(62.8%)	
Female n, %	92(29.6%)	207(37.2%)	
Age (years old)	49.8 ± 11.3	59.4 ± 9.8	< 0.001*
BMI(Kg/m^2^)	25.4 ± 3.7	24.7 ± 3.5	0.006*
Waist circumference(cm)	85.9 ± 10.1	84.7 ± 10.5	0.08
Total cholesterol(mg/dl)	191.2 ± 34.5	198.2 ± 36.2	0.005*
HDL cholesterol(mg/dl)	54.4 ± 14.9	57.8 ± 15.8	0.002*
Triglyceride(mg/dl)	136.8 ± 83.8	129.2 ± 85.2	0.205
LDL cholesterol(mg/dl)	110.0 ± 31.5	115.3 ± 32.7	0.021*
Peptic ulcer n, %	111(35.7%)	240(43.2%)	0.032*
GERD n, %	69(22.2%)	118(21.2%)	0.741
*H. pylori* infection n, %	124(39.9%)	257(46.2%)	0.071

*Indicates a significant difference, *p* < 0.05

*BMI* body mass index, *LDL* low-density lipoprotein, *HDL* high-density lipoprotein, *BMD* bone mineral density, *GERD* gastro-esophageal reflux disease, H. pylori *Helicobacter pylori*

### Simple and multiple stepwise logistic regression analyses of variables associated with decreased BMD

Simple logistic regression (Table 3) showed decreased BMD was significantly associated with the female gender (OR 0.71, 95% CI 0.53–0.94, *P* = 0.023), advanced age (OR 1.09, 95% CI 1.07–1.10, *P* < 0.001), low BMI (OR 0.95, 95% CI 0.91–0.99, *P* = 0.006), total cholesterol (OR 1.01, 95% CI 1.00–1.01, *P* = 0.005), low-density lipoprotein (LDL) cholesterol (OR 1.01, 95% CI 1.00–1.01, *P* = 0.021), high-density lipoprotein (HDL) cholesterol (OR 1.02, 95% CI 1.00–1.02, *P* = 0.002), and peptic ulcer disease (OR 1.37, 95% CI 1.03–1.82, *P* = 0.032). The multivariate analysis that was conducted with these risk factors revealed that only advanced age (OR 1.09, 95% CI 1.08–1.11, *P* < 0.001), and low BMI (OR 0.91, 95% CI 0.87–0.95, *P* < 0.001) were independently significantly associated with decreased BMD. The *H. pylori* infection was not independently significantly associated with decreased BMD (OR 1.30, 95% CI 0.98–1.71, *P* = 0.071).

**Table 3:** Regression analysis for association of decreased BMD with different variables

Variables	Simple logistic regression OR (95% CI)	p value	multiple stepwise logistic regression OR (95% CI)	*p* value
Sex	0.71(0.53–0.94)	0.023*		
Age	1.09(1.07-1.10)	< 0.001*	1.09(1.08-1.11)	< 0.001*
BMI	0.95(0.91-0.99)	0.006*	0.91(0.87-0.95)	< 0.001*
Waist circumference	0.99(0.98–1.00)	0.08		
Total cholesterol	1.01(1.00–1.01)	0.005*		
HDL cholesterol	1.02(1.00–1.02)	0.002*		
TG	1.00(0.99-1.001)	0.207		
LDL cholesterol	1.01(1.00–1.01)	0.021*		
Peptic ulcer	1.37(1.03–1.82)	0.032*		
GERD	0.95(0.68-1.32)	0.74		
H. pylori infection	1.30(0.98–1.71)	0.071		

*Indicates a significant difference, *p* < 0.05

*BMI* body mass index, *LDL*, low-density lipoprotein, *HDL* high-density lipoprotein, *BMD* bone mineral density, *GERD* gastro-esophageal reflux disease, H. pylori *Helicobacter pylori*

Because the confounding effect of age was strong with nonrandomized assignment, propensity score-matching was used for age adjustment. The 234 well-balanced pairs of participants, with a 1:1 ratio after propensity score matching of age, were evaluated for risk factor of decreased BMD. In these propensity score-matched participants, the mean age was 53.3 ± 10.35 years in the normal BMD group and 53.4 ± 10.33 years in the decreased BMD group. There was no significant difference in age between the two groups (*p* = 0.961). The covariates of these well-balanced pairs of participants were conducted for simple and multiple stepwise logistic regression analysis. In simple logistic regression, the BMI (OR 0.92, 95% CI 0.87–0.97, *P* = 0.001), waist circumstance (OR 0.98, 95% CI 0.96–0.99, *P* = 0.006) and *H. pylori* infection (OR 1.60, 95% CI 1.11–2.30, *P* = 0.012) were significantly associated with decreased BMD. In multivariate analysis, *H. pylori* infection (OR 1.62, 95% CI 1.12–2.35, *P* = 0.011) and BMI (OR 0.92, 95% CI 0.87–0.97, *P* = 0.001) were independent significant risk factors of decreased BMD without confounding effect of age (Table 4).

**Table 4:** Regression analysis for association between decreased BMD and different variables after propensity score matching

Variables	Simple logistic regression OR (95% CI)	*p* value	Multiple stepwise logistic regression OR (95% CI)	*p* value
Sex	0.80(0.54–1.19)	0.27		
BMI	0.92(0.87–0.97)	0.001*	0.92(0.87-0.97)	0.001*
Waist circumference	0.98(0.96–0.99)	0.006*		
Total cholesterol	1.00(0.99–1.01)	0.257		
HDL	1.01(1.00–1.02)	0.082		
TG	1.00(0.99–1.001)	0.672		
LDL	1.00(0.99–1.01)	0.425		
Peptic ulcer	1.10(0.75–1.60)	0.632		
GERD	0.88(0.56–1.38)	0.564		
H. pylori infection	1.60(1.11–2.30)	0.012*	1.62(1.12-2.35)	0.011*

*Indicates a significant difference, *p* < 0.05

*BMI* body mass index, *LDL* low-density lipoprotein, *HDL* high-density lipoprotein, *BMD* bone mineral density, *GERD* gastro-esophageal reflux disease, H. pylori *Helicobacter pylori*

## Discussion

The main finding of this retrospective cross-sectional study revealed that advanced age and low BMI were significant risk factors of decreased BMD in the nonrandomized assignment. In addition, *H. pylori* infection was significantly associated with decreased BMD in selected propensity score-matched participants with respect to age. In other words, *H. pylori* infection was a risk factor of decreased BMD without the confounding effect of age, because the age corresponded to an increase in risk of osteoporosis [2].

This result was compatible with the past studies regarding the association between *H. pylori* infection and decreased BMD [10, 12, 14]. Several possible mechanisms might explain this finding. First, *H. pylori* infection may result in chronic gastritis and induce systemic inflammation with the release of cytokines, including tumor necrosis factor- 훼, interleukin-1 and interleukin-6 [15]. These inflammatory cytokines are known to result in bone resorption, so *H. pylori* infection may affect bone turnover indirectly [16]. The second mechanism was that chronic *H. pylori* infection might cause the gastric mucosal atrophy which would decrease acid secretion. The hypochlorhydric stomach affects calcium absorption, calcium homeostasis and bone mass [17]. In addition, the low serum vitamin B12 level was found in the patients with *H. pylori* infection [18]. If the serum vitamin B12 levels are low, the folate becomes trapped as methyltetrahydrofolate and then interrupts for folate-related DNA synthesis. This reaction is an important factor of bone remodeling, so the low level of vitamin B12 may result in decreased BMD [19].

Besides, *H. pylori* infection was treated with the eradication therapy as triple or quadruple regimen, such as a proton pump inhibitor, clarithromycin, amoxicillin or tetracycline, and metronidazole, with or without bismuth. One study described cytokine gene expression as significantly decreasing after *H. pylori* was eradicated [20]. In a meta-analysis, mucosal atrophy of the stomach was improved after successful eradication therapy [21]. Because the cytokines result in bone resorption and gastric mucosal atrophy affects calcium metabolism, the improvement of gastric mucosa and decreased inflammatory cytokine by successful eradication therapy might be able to decrease the incidence of osteoporosis. Two studies support this hypothesis. Hong-Mo Shih et al. reported the incidence of osteoporosis relatively reduced in early eradication of *H. pylori* group, compared to the late eradication group by an analysis from the National Health Insurance Database in Taiwan [22]. In Japan, the success of *H. pylori* eradication may contribute to decrease the risk of osteoporosis. In our study, we found that *H. pylori* infection was independently significantly associated with decreased BMD after age adjustment, but eradication of *H. pylori* was not recorded in this health examination database. A further prospective cohort study is necessary to confirm that BMD would be improved after triple or quadruple *H. pylori* eradication therapy.

Advanced age and low BMI are well-known risk factors of osteoporosis and bone fracture [2]. From the National Health and Nutrition Examination Survey (NHANES) 2005–2006 in US, 49% of US women age 50 years and older had decreased BMD, and 30% of older men had decreased BMD [23]. The prevalence rates of BMD from Nutrition and Health Survey in Taiwan 2005–2008 was higher in advanced age. In females, the prevalence rate was 50.3% in the 60 years and older group, and increased to 63.7% in the 70 years and older group. In male, the prevalence rate was 18.6% in the 60 years and older group, and increased to 45.4% in those aged 70 years and older [24]. One research showed that the weight loss appears to increase the rate of hip bone loss, even in obese men undergoing voluntary weight reduction [25]. This present study reported a similar result where the advanced age and low BMI had the strong association with the decreased BMD.

Several researches have reported that the GERD was associated with osteoporosis. However, the vertebral fractures or kyphosis were also found in these studies [26–28]. In the present study, the GERD was not significantly associated with decreased BMD. Because this study was derived from the health examinations, these subjects may be healthy relatively without any bone deformities. Besides, two studies revealed peptic ulcer disease was an independent risk factor for osteoporosis [6, 29]. The mechanism of this association was not clear. The malabsorption of calcium and macroelements due to the defective stomach and duodenal epithelium in which inflammation at these sites results may play a role in bone metabolism. Our result showed the peptic ulcer was not a risk factor of decreased BMD, but it tended to be associated with decreased BMD in univariate logistic regression analysis. The disorder of the small intestine may affect the absorption of these substances more than disorders of the stomach and duodenum.

There were several limitations in our study. First, our study was conducted in a single hospital, which might not be representative of other settings. Second, retrospective studies based on abstraction of medical records are constrained by the accuracy and completeness of such records. Third, some laboratory data, including serum calcium, serum phosphorus, serum specific alkaline phosphatase or serum vitamin D level were unavailable, because this study was retrospective from the health examination.

## Conclusions

In conclusion, advanced age and low BMI were independent significant risk factors of decreased BMD in this retrospective study. Besides low BMI, *H. pylori* infection was independently significantly associated with decreased BMD in selected propensity score-matched participants with respect to age. Further prospective cohort studies including the potential important factors are required to confirm this association in the Taiwan population.

## Abbreviations

BMD: Bone mineral density
BMI: Body mass index
CI: Confidential interval
CLO test: Campylobacter-like organism test
DEXA: Dual energy X-ray absorptiometry scan
GERD: Gastro-esophageal reflux disease
*H. pylori*: *Helicobacter pylori*
HDL: High-density lipoprotein
LDL: Low-density lipoprotein
